# *Caenorhabditis elegans*: a model to monitor bacterial air quality

**DOI:** 10.1186/1756-0500-4-503

**Published:** 2011-11-18

**Authors:** Cécile Duclairoir Poc, Anne Groboillot, Olivier Lesouhaitier, Jean-Paul Morin, Nicole Orange, Marc JG Feuilloley

**Affiliations:** 1 Laboratory of Microbiology-Signals and MicroEnvironment, Normandy University, University of Rouen, EA 4312, 55 rue Saint Germain, 27000 Evreux, France; 2 U644, Faculty of Medecine and Pharmacy, 22 boulevard Gambetta, INSERM Normandy University, University of Rouen, 76181 Rouen cedex, France

## Abstract

**Background:**

Low environmental air quality is a significant cause of mortality and morbidity and this question is now emerging as a main concern of governmental authorities. Airborne pollution results from the combination of chemicals, fine particles, and micro-organisms quantitatively or qualitatively dangerous for health or for the environment. Increasing regulations and limitations for outdoor air quality have been decreed in regards to chemicals and particles contrary to micro-organisms. Indeed, pertinent and reliable tests to evaluate this biohazard are scarce. In this work, our purpose was to evaluate the *Caenorhaditis elegans *killing test, a model considered as an equivalent to the mouse acute toxicity test in pharmaceutical industry, in order to monitor air bacterial quality.

**Findings:**

The present study investigates the bacterial population in dust clouds generated during crop ship loading in harbor installations (Rouen harbor, Normandy, France). With a biocollector, airborne bacteria were impacted onto the surface of agar medium. After incubation, a replicate of the colonies on a fresh agar medium was done using a velvet. All the replicated colonies were pooled creating the "Total Air Sample". Meanwhile, all the colonies on the original plate were isolated. Among which, five representative bacterial strains were chosen. The virulence of these representatives was compared to that of the "Total Air Sample" using the *Caenorhaditis elegans *killing test. The survival kinetic of nematodes fed with the "Total Air Sample" is consistent with the kinetics obtained using the five different representatives strains.

**Conclusions:**

Bacterial air quality can now be monitored in a one shot test using the *Caenorhaditis elegans *killing test.

## Background

The deep impact on health from chemical pollutants [1] is now a main concern of governmental policies in most countries. As well as airborne nanoparticles and chemicals [2], airborne bacterial and fungal contaminants are also of major importance in human health [3,4]. These micro-organisms may lead to typical respiratory tract infections but also to delayed sensitization (allergic) reactions somehow considered as the "*epidemy of the 21st century*" [5]. Some of these micro-organisms simply originate from the environment but human activities are also important sources of aerial biological contaminants [6]. It would be particularly interesting to develop a microbial air quality alert system equivalent to that for chemical pollutants. Microbial air quality is commonly evaluated by measuring the global concentration in the air of bacteria and fungi by culture-dependant or bio-molecular methods [7]. High microbial concentrations in the air are generally associated to potential danger. The search for endotoxins has been developed [8]. The composition of microbial communities is often assessed by molecular methods [8,9] and pathogen species have been also detected by PCR with specific probes [10]. Studying airborne bacterial and fungal communities in land [4,9], urban [11-13], and occupational [14] environments now allows an almost complete description of environmental air micro-organisms biodiversity. However, the identification of microbial species and strain is not sufficient to extrapolate at the sanitary risk level, as many different parameters may affect virulence. Indeed, both contact mediated and toxin dependent virulence are regulated by environmental conditions including temperature [15], bacteriophages [16] and of course microbial communication factors [17]. Sometimes bacterial strains are generally considered safe and devoid of pathogenic potential and, however, induce clinical infections [18,19]. Determining the real risk associated to airborne micro-organisms first requires collecting a maximum amount of micro-organisms in conditions which avoid the stress or even the destruction of the most sensitive micro-organisms which are usually the most metabolically active [20]. The identification of the composition of this population should be realized in a second step but the essential point is to evaluate the virulence of the complex community. This requires a cheaper and more rapid test than classical animal assays but also less sensitive than *in vitro *cytotoxicity tests. In the present study, the chosen model was the *Caenorhabditis elegans *worm. It is a versatile metazoan model previously used to assess of the virulence of many human pathogens [21,22], and especially to study primary respiratory chain dysfunction in humans [23]. Spontaneous predation and ingestion of bacteria provoke nematode death by contact dependent bacterial virulence and by the bacterial secretion of toxins [21,22]. Thus, in the present study, we investigated the risk linked to airborne bacteria in the dust cloud generated during crop ship loading in harbor installations located in close proximity of residential areas. After collection of the total microbial population, bacteria were identified by traditional phenotypic and molecular techniques. The virulence of some individual representatives chosen among the collected bacteria was compared using the nematode *C. elegans*. to that of the pooled replicated bacterial population defined as "Total Air Sample".

## Methods

### Aerial bacteria collection

Bacteria were collected in January 2009 at the dockside of Rouen harbour (Normandy, France) during loading from crop silo onto ship from stocking silo. All operations were done in the morning. Samples were obtained by aspiration of 30, 60, 90, or 190 L (under a flow rate 100 L.min^-1^) in the dust cloud of particles generated by crop transfer using an AirTest Omega Biocollector (LCB, France). In this device, air is drawn in through a 0.5 mm grid to remove macroscopic particles and the air flow is directed towards the impact medium where micro-organisms are trapped. In order to limit the stress on bacteria, a semi-solid impact medium, Tryptipcase Soy Agar (TSA), was made with Tryptic Soy Broth (TSB) diluted 1:5 containing agar (10 g.L^-1 ^) and amphotericin B (25 mg.L^-1^) as a fungicide.

**Figure 1:**
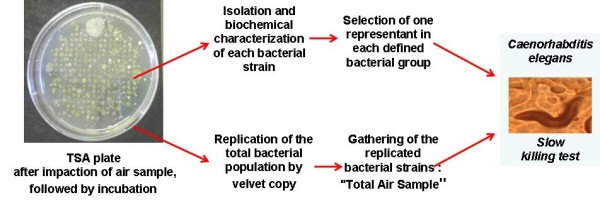
**Plotted experimental strategy from collected airborne bacteria to the evaluation of their virulence**.

After collection, impacted TSA plates, presented in Figure 1, were incubated at 30°C for 72 h to reveal the colonies of cultivable bacteria. Agar plates showing well separated and countable colonies were selected and before other manipulation, a velvet copy of these plates was done. This velvet was laid on a new TSA plate which afterwards was incubated at 30°C for 72 h. All the formed colonies were transferred to and grown in the same culture in 1:5 diluted TSB and then frozen at - 80°C with a final glycerol concentration of 30%. This mixture of strains collected from the air impaction was designed as "Total Air Sample". Subsequently, from the original impacted TSA plate, individual colonies were removed and isolated as pure cultures on TSA for further analysis and storage at - 80°C as previously described.

### Bacterial characterization and identification

Each isolated bacterial strain was initially characterized by morphological observation (colony aspect, cell shape, motility, endospore), Gram staining and biochemical tests (oxidase, catalase and oxidative metabolism). These observations allowed different bacterial groups to be defined. In each group including potential pathogens, one representative strain was chosen and submitted to further characterization on API tests galleries. API kits were operated according to the manufacturer's instructions (BioMérieux, France). For Gram-positive bacteria, API 50CH was used for the identification of *Bacillus*, API ID STAPH 32 for identification of germs of the genus *Staphylococcus *or *Microccocus *and API Coryne for Gram-positive catalase-positive non-sporing rods. For Gram-negative bacteria, API 20E was used for the identification of *Enterobacteriaceae *and API 20NE for non fermenting rods. Species identification of representative strains was confirmed by 16S RNA sequencing. For amplification of the complete 16S RNA gene, universal primers UNI_OL (5¢-AGAGTGTA GCGGTGAAATGCG-3¢,) and UNI_OR (5¢-ACGGGCGGTGTGTACAA-3¢,) were used as suggested by Sauer et *al*. [24]. Amplicons were then purified through migration on agarose gel and sequenced directly by using the amplification primers UNI_OL and UNI_OR (Qiagen, Germany). Afterwards 16S RNA fragments (+/- 750 pb) were analyzed (Qiagen, Germany) and homologies with sequences of other eubacteria were determined by searching the NCBI (http://www.ncbi. nlm.nih.gov/sutils/genom_table.cgi) and RDP (http://rdp.cme.msu.edu/seqmatch/seqmatch_intro.jsp) data banks using BLAST software. An isolate was positively identified when the full-length 16S RNA gene yielded a >98.3% sequence similarity with the closest bacterial species registered in the data banks.

### Evaluation of bacterial virulence using the *Caenorhabditis elegans *killing tests

The virulence of the airborne bacterial population, i.e. the "Total Air Samples", was compared to that of individual representatives of bacterial strains collected in the air using the slow killing tests on the nematode *Caenorhabditis elegans*, accordingly Figure 1.

Experiments were conducted using the wild-type Bristol strain N2 of *C. elegans *provided by the *Caenorhabditis *Genetics Center (Minneapolis, MN, USA). Worms were maintained under standard culturing conditions at 22°C on nematode growth medium (NGM) containing 3 g NaCl, 2.5 g peptone, 17 g agar, 5 mg cholesterol, 1 mL 1 M CaCl_2_, 1 mL 1 M MgSO_4_, 25 mL 1 M KH_2_PO_4_, H_2_O for 1 L of medium. This medium was plated on Petri dishes and *Escherichia coli *OP50 as added as a normal food source [21]. For virulence tests, synchronized worms of the same development level were obtained by bleaching an adult population using sodium hypochlorite/sodium hydroxide solution [25]. The resulting eggs were incubated at 22°C on *E. coli *OP50 lawns until the worms reached the L4 life stage (48 h). The stage of development was confirmed by microscopic observation.

For bacterial virulence assays, "Total Air Samples" or individual air strain aliquots standardized by dilution (at OD_580 _= 1) were prepared by spreading 100 μL on 35 mm Petri dishes containing NGM supplemented with 0.05 mg.mL^-1 ^5-fluoro-2'-deoxyuridine (FUDR), a eukaryote DNA synthesis inhibitor preventing *C. elegans *egg offspring during the experiments. Plates were incubated overnight at 29°C and then transferred at room temperature for 4 h. In each Petri dish a mean of 20 L4 synchronized worms, harvested in M9 solution (3 g KH_2_PO_4_, 6 g NaHPO_4_, 5 g NaCl, 1 mL 1 M MgSO_4_, H_2_O in 1 L) were layered. Plates were incubated at 22°C and worm survival was scored every 24 h throughout a 22 days period using an Axiovert S100 optical microscope (Zeiss, Oberkochen, Germany) equipped with a Nikon digital Camera DXM 1200F (Nikon Instruments, Melville, NY, USA). A worm was considered dead when it remained static without grinder movements for 20 s. Results are expressed as percentage of worms surviving every day and were calculated as the mean of 3 independent assays in which each point was the average of 3 replicates. Nematode survival was calculated using the Kaplan-Meier method, and survival differences were tested for significance using the log-rank test (GraphPad Prism version 4.0; GraphPad Software, San Diego, California, USA).

## Results and Discussion

### Identification of airborne bacteria in the dust cloud generated by crop transfer

Plates obtained with 30 L air presented a sufficient number of colonies which remained well separated. In cultures obtained with higher air volumes, bacterial colonies were too numerous for correct isolation.

As seen Figure 1, velvet replicates of impacted TSA plates were realized to obtain the "Total Air Sample". A total of 323 bacterial strains were isolated. The corresponding calculated bacterial concentration in the air was about 1.1 × 10^4 ^CFU.m^-3 ^as shown in Table 1. This bacterial concentration in the dust cloud generated by crop loading was slightly higher than obtained in urban air, which ranged from 5.5 × 10^2 ^to 2.5 × 10^3 ^CFU.m^-3 ^[7,11]. The large quantity of dusts and particles, which represents the principal support of bacteria in the air [26] can explain this result. It is also interesting to note that the values of bacterial load measured at the centre of the dust cloud are markedly lower than previously measured [27]. This difference should be attributed to the application of more recent procedures aimed at reducing particle emission during ship loading (for instance covered conveyor belts).

**Table 1:** Airborne bacterial composition in the dust cloud resulting from crop transfer

Sample		Next to crops ship loading winter 2009	
**Collected volume**		**30L**	

**Incubation conditions**		**30°C on 1:5 diluted TSA plate**	

**UFC/air m**^**3**^		**10767**	

	**Group 1**		17.6%
			
**Gram-negative rods**	**Group 2**	72.4%	4.0%
			
	**Group 3**		39.9%
			
	**Group 4**		10.8%
			
**Gram-positive rods**	**Group 5**	16.4%	5.3%
			
	**Group 6**		11.1%
			
**Gram-positive cocci**	**Group 7**	6.5%	3.7%
			
	**Group 8**		2.8%
			
**unexploitable strains**		4.6%	

Eight groups were represented: Gram-negative oxidase-negative strictly aerobic rods such as *Acinetobacter, Pseudomonas*... (Group 1), Gram-negative oxidase-positive facultatively anaerobic rods such as *Aeromonas*... (Group 2), Gram-negative oxydase-negative facultatively anaerobic rods mainly *Enterobacteriaceae *(Group 3), Gram-negative oxidase-positive strictly aerobic rods like *Pseudomonas*...(Group 4), Gram-positive sporing rods such as *Bacillus*...(Group 5), Gram-positive catalase-positive nonsporing rods such as *Arthrobacter*... (Group 6), Gram-positive catalase-positive strictly aerobic cocci such as *Micrococcus*... (Group 7), Gram-positive catalase-positive facultatively anaerobic cocci such as *Staphylococcus*... (Group 8).

Morphological characters, Gram staining and biochemical tests were carried out for the 323 bacterial strains. These orientation tests allowed to distribute the bacterial population in 8 groups, *i.e*. Gram-negative oxidase-negative strictly aerobic rods such as *Acinetobacter, Pseudomonas*... (Group 1), Gram-negative oxidase-positive facultatively anaerobic rods such as *Aeromonas*... (Group 2), Gram-negative oxydase-negative facultatively anaerobic rods mainly *Enterobacteriaceae *(Group 3), Gram-negative oxidase-positive strictly aerobic rods like *Pseudomonas*...(Group 4), Gram-positive sporing rods such as *Bacillus*...(Group 5), Gram-positive catalase-positive nonsporing rods such as *Arthrobacter*... (Group 6), Gram-positive catalase-positive strictly aerobic cocci such as *Micrococcus*... (Group 7), Gram-positive catalase-positive facultatively anaerobic cocci such as *Staphylococcus*... (Group 8). No Gram-positive catalase-negative were found. Gram-negative rods accounted for the greatest part of the bacterial population (72.4%) with the following distribution: 39.9% for Group 3, 17.6% for Group 1, 10.8% for Group 4 and 4.0% for Group 2. Concerning Gram-positive bacteria, the predominant group corresponded to Group 6 (11.1%) followed by Group 5 (5.3%), Group 7 (3.7%), and Group 8 (2.8%). The bacterial composition, characterized by a predominance of Gram-negative bacilli, appeared unchanged compared to a similar study [27]. This observation is logical with regards to the nature of the material at the origin of the particles (crops). The main bacterial group, i.e. *Enterobacteriaceae*, Group 3, (39.9%), corresponds to bacterial species frequently associated with plants, and behave as epiphyte, endophyte or even pathogens [28]. These bacteria colonize crops during field growth, survive and even proliferate during storage and then are present in the dust cloud [29]. The second group of bacteria in quantity is Group 1, represented by *P. syringae*, a germ present in the phyllosphere and generally adapted to growth at low temperature [30]. Catalase-positive non sporulated Gram-positive rods, Group 6, representing 11.1% of bacteria in the dust cloud, should correspond to bacteria of the genus *Corynebacteria, Rhodococcus *or *Arthrobacter *also usually present in the ground and already detected in urban air [11] and during crop manipulations [29].

The aim of this study was to monitor the bacterial air quality, and the study was focused on the groups including potential pathogens: Group 1, 3-5, and 8. From each of these groups, a representative strain was randomly chosen among the isolated strains. They were submitted to API gallery identification and 16S RNA sequencing. As shown in Table 2, all the orientation results were confirmed by those of the identification. Indeed, *P. syringae *is an oxidase-negative *Pseudomonas*, then it was pooled with *Acinetobacter*. *Brevundimonas *is related to the genus *Pseudomonas*. The correlation between the API gallery identification and 16S RNA is quite good at the genus level.

**Table 2:** Identification by API galleries and 16S RNA sequencing of the five representatives: *P. syringae, S. marcescens, Brevundimonas, B. cereus*, and *S. aureus*

	API Test	16S RNA identification
***Group 1***	*Pseudomonas luteola (G)*	*Pseudomonas syringae*

***Group 3***	*Serratia liquefaciens (VG)*	*Serratia marcescens*

***Group 4***	*Brevundimonas vesicularis*	*Brevundimonas *sp.

***Group 5***	*Bacillus cereus (Exc)*	*Bacillus thuringiensis, B. cereus, or B. anthracis*

***Group 8***	*Staphylococcus sciuru (Exc)*	*Staphylococcus aureus*

The discrepancies between identification results, especially at the species level, could be explained by the incomplete covering of clinical API galleries for the identification of environmental bacteria. This imprecision could also result from the 16S RNA strategy of identification, especially when it is applied over a large diversity of environmental bacteria whose compilation is not complete in data bases such as NCBI/PUBMED.

### Comparison of population and bacterial virulence using the *Caenorhabditis elegans *killing test

This study deals with the impact of bacteria present in outdoor air on human health. A robust and practical experimental model was therefore needed in relation to respiratory diseases resulting from bacterial infection. The nematode *C elegans *model is known to model mammalian bacterial pathogenesis [21] and especially in human respiratory dysfunction [23].

**Figure 2:**
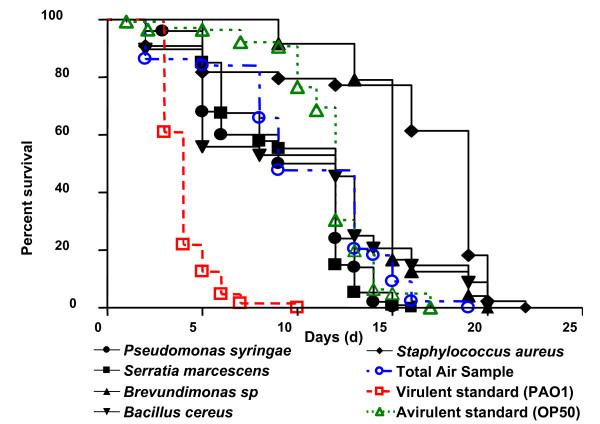
**Kinetics of survival of *C. elegans *exposed to bacteria**. Kaplan-Meier survival plots of worms fed with the opportunistic pathogen *Pseudomonas aeruginosa *PAO1, bacterial strains isolated from air: *Pseudomonas syringae *(Group 1 *: Acinetobacter*...), *Pantoae sp *(Group 3: *Enterobacteriaceae*...), *Brevundimonas sp *(Group 4 *Pseudomonas*...), *Bacillus cereus *(Group 4: *Bacillus*...), and *Staphylococcus aureus *( Group 8: *Staphylococcus*...) and the non-virulent strain *Escherichia coli *OP50, and "Total Air Sample". Experiments were done in triplicate.

The practical experimental advantages of this free-living worm are its ability to feed solely on bacteria, its short life cycle, and its easy cultivation in large number [31]. The survival kinetics of *C. elegans *in the presence of each bacterial representative (*P. syringae, Serratia marcescens, Brevundimonas, B. cereus*, and *S. aureus*) was studied for a maximum of 22 days (Figure 2). Even in the presence of their normal food source (*Escherichia coli *OP50) worms progressively die of age, all died after 17 days of culture. It is important to remember that the DNA synthesis inhibitor (FUDR) present in the medium prevents *C. elegans *egg offspring and population renewal. The survival of worms in the presence of these bacteria was compared to the one measured using the opportunistic pathogen *P. aeruginosa *PAO1, frequently responsible for respiratory tract infections [32]. Worms died rapidly in the presence of *P. aeruginosa *PAO1 (10 days) and none of the environmental strains collected in the air had equivalent virulence. In fact, all the survival kinetics were highly significantly different of those obtained in the presence of *P. aeruginosa *PAO1 (P < 0.0001 log-rank test). *P. syringae, S. marcescens*, and *B. cereus *presented against *C. elegans *increased or similar virulence compared to *E. Coli *OP50.

Surprisingly, two strains: *Brevundimonas sp*., and *S. aureus *appeared less virulent than *E. Coli *OP50 (P < 0.0001 log-rank test). Even if *S. aureus *is accepted as usually pathogen and *Brevundimonas *considered as an opportunistic pathogen, we have to keep in mind that the bacterial virulence depends on the strains and is closely related to the bacterial adaptation to its microenvironment. But from the random selection of the representatives, no strain is known as strictly human pathogen. Also, according *C. elegans *test, no sanitary threat appeared because all representative strains were less virulent than the standard opportunistic pathogen *P. aeruginosa *PAO1. As isolation of all the bacterial spots on the impacted plate is rather fastidious work and complex populations should have virulence which differs from individual strains. Another approach is to test the virulence of the "global" impacted bacterial population against *C. elegans*, as shown in Figure 1. This was done with the bacterial mix, defined as "Total Air Sample". As with the representatives, virulence against nematodes was studied for the "Total Air Sample", corresponding to the bacterial population collected during crop loading on ships (plotted in Figure 2). The kinetics of the survival of worms with "Total Air Samples" was surrounded by most of the representative plots and was significantly different from that of *P. aeruginosa *PAO1 (P < 0.0001 log-rank test). Moreover, within the first 11 days of the study, the percentage of death of the worm population was greater using "Total Air Samples" than the avirulent standard *E. coli *OP50. This difference vanished afterwards and in both cases all worms were dead between 17 and 19 days.

**Figure 3:**
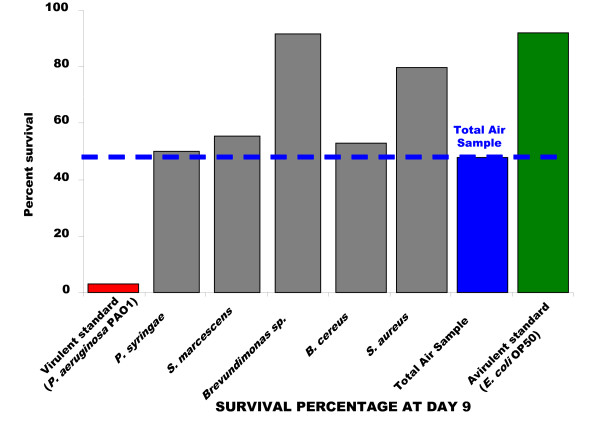
**Comparison of the survival of worms exposed to bacteria at Day 9**. Experiments were done in triplicate.

The comparison of the lethal effects of the "Total Air Sample" with each environmental representative was clearly illustrated at Day 9 (Figure 3). This time corresponds to the last day of nematodes survival in the presence of *P. aeruginosa *PAO1. The survival of *C. elegans *exposed to *P. syringae *(50.00%), *Pantoae spp *(55.26%), and *B. cereus *(52.11%) was in the same range as for worms fed with "Total Air Sample" (47.73%). Nevertheless, as noted at Day 9, *S. aureus *and *Brevundimonas *favored the survival of nematodes with 79.55% and 91.66% of survival percentage respectively, compared to 47.73% for "Total Air Sample". Thus, nematode survival in the presence of the "Total Air Sample" seems pertinent to represent all bacteria synergies and individual virulence. The "Total Air Sample" can be considered without great sanitary risk for all healthy and non immuno-depressed people, as each representative strain.

Thence, any environmental sample with the help of a biocollector makes the assessment of the bacterial sanitary risk possible. After incubation of the softed impacted TSA plates, the total and cultivable bacterial population collected can directly feed the *C. elegans *nematode model, whose latest death shows the good quality and bacterial safety of the sample environment.

## Conclusion

This study establishes that the outdoor air quality can be evaluated with the help of *C. elegans *nematodes alternative model for bacterial virulence aspects, even with high concentrations of airborne bacteria. Without the fastidious microbiological labor (isolation and bacterial identification), air quality can be monitored easily with a one-shot virulence test of the collected bacterial population obtained from a TSA plate impacted by outdoor air.

## Competing interests

The authors declare that they have no competing interests.

## Authors' contributions

CDP and AG conceived the study, designed and carried out some of the experiments and drafted the manuscript. OL participated in the design of the study and carried out some of the experiments. JPM encourage the use of *C. elegans *in air assessment. NO led the global project design and coordinated it. MF coordinated toxicological aspects. All authors red and approved the final manuscript.
